# Defining the role of TT-TG and TT-PCL in the diagnosis of lateralization of the Tibial tubercle in recurrent patellar dislocation

**DOI:** 10.1186/s12891-020-03900-3

**Published:** 2021-01-08

**Authors:** Peng Su, Nengri Jian, Beini Mao, Zhong Zhang, Jian Li, Weili Fu

**Affiliations:** 1 Department of Orthopaedic Surgery, West China Hospital, Sichuan University, 37 Guoxue lane, Wuhou District, Chengdu, China; 2 Department of Radiology, West China Hospital, Sichuan University, 37 Guoxue lane, Wuhou District, Chengdu, China

**Keywords:** Recurrent patellar dislocation, Computed tomography, Magnetic resonance imaging, Tibial tubercle–trochlear groove, Tibial tubercle–posterior cruciate ligament

## Abstract

**Background:**

The radiological indicators can help doctors determine whether to make tibial tubercle transfer. But which indicator is better is still in question.

**Methods:**

117 knees in 103 patients who had undergone patellar surgery and 60 knees in 58 patients who had no history of patellar dislocation from 2014 to 2019 were analyzed. Significant differences of tibial tubercle–trochlear groove (TT-TG) on CT and tibial tubercle–posterior cruciate ligament (TT-PCL) on MRI between the case group and the control group were estimated by an unpaired t test. Significant differences between TT-TG on CT and TT-TG on MRI were estimated by a paired t test. The correlation between TT-PCL on MRI and tibial width was estimated by Pearson test. Receiver operating characteristic (ROC) curves and the area under the ROC curve (AUC) were measured to assess the diagnostic accuracy of TT-TG and TT-PCL on MRI.

**Results:**

The intraclass correlation coefficient (ICC) for TT-TG between CT and MRI evaluated by two raters was were 0.566. When comparing TT-TG on CT with that on MRI, the mean difference was 2.5 mm (*p*< 0.001). The mean TT-TG difference on CT between the case group and the control group was 5.3 mm, which was significantly bigger than the mean TT-PCL difference on MRI of 1.2 mm(*p<* 0.001). AUC of TT-TG on CT and TT-PCL were 0.838 and 0.580 (*P*< 0.001). TT-PCL correlated with tibial width (*r=*0.450, *P<* 0.001).

**Conclusion:**

A statistically significance and a fair ICC proved that TT-TG could not be used interchangeably. The bigger mean difference between the case group and the control group and better AUC proved that TT-TG on CT might be an indicator more suitable for measuring the lateralization of the tibial tubercle. And TT-PCL should be considered as an individual parameter because of the significant correlation between TT-PCL and tibial width.

## Background

Recurrent patellar dislocation is a common disease, especially in adolescents and young adults whose disease rate can be 29 per 100,000 [1]. Many factors can contribute to recurrent patellar dislocation, and surgeons need to select surgical techniques which range from soft tissue surgery to bony correction. Bony procedures include trochleoplasty and medial or distal tubercle transfer. Soft tissue procedures include medial patellofemoral ligament (MPFL) reconstruction and lateral release [2]. For surgeons, whether to do tibial tubercle transfer mainly is determined by the extent of lateralization of tibial tubercle. This is most commonly assessed by TT-TG which is the distance between the anterior tibial tubercle (TT) and the deepest point of the trochlear groove (TG). Goutallier et al. firstly described it on an axial radiograph in 1978 [3]. Subsequently, Dejour measured the TT-TG on CT, making the measurement more precise [4, 5].

The cutoff of 20 mm was considered pathological and as an indication for surgery in recurrent patellar dislocation [5, 6]. Recently, TT-TG was measured on MRI because of reduced radiation exposure for patients. But The interchangeability of TT-TG between CT and MRI was in controversy. Shoettle et al. argued that the measurement could be used interchangeably between CT and MRI [7]. On the contrary, Anley argued that the measurements for the TT-TG cannot be used interchangeably between CT and MRI. And the cutoff value for TT-TG on CT should not be applied to MRI [8].

In recent years, some literatures proved that the measurement of TT-TG could be influenced by the flexion of the knee, For eliminating such effect, Seitlinger et al. proposed a new measurement-TT-PCL which was defined as the distance between the midpoint of the insertion of the patellar tendon and the medial border of the PCL, and recommended 24 mm as the cutoff value [9]. But Boutris advocated the use of a new pathologic TT-PCL threshold of 21 mm [10]. Except for TT-PCL, Tensho proposed TT-PCL ratio which was calculated as the TT-PCL distance divided by the tibial width in order to adjust for individual differences [11]. This indicated that the TT-PCL distance might be influenced by the tibial width.

The aim of our study was to determine: 1) TT-TG or TT-PCL, which one was more effective in evaluating patellar displacement. 2) confirm the correlation between the TT-PCL and the width of the tibial plateau.

## Methods

**Fig. 1:**
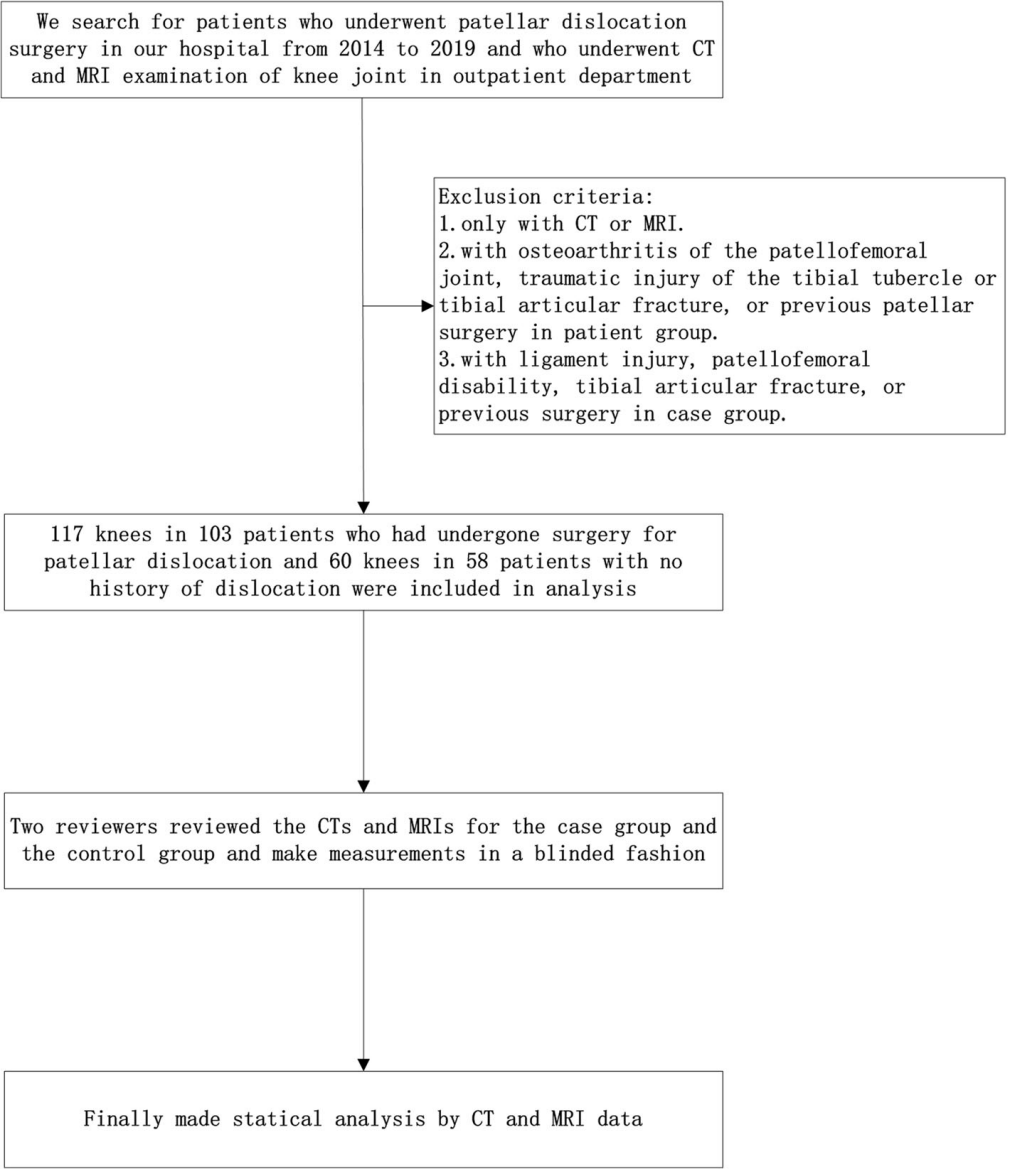
The flow chart of the research

Before the research was started, it was approved by the hospital ethics committee. The imaging data including CT and MRI of 161 patients between 2014 and 2019 was reviewed. The case group consisted of 103 patients (117 knees, age: 19.2±5 years old, female/male: 75/28). They all underwent surgery for recurrent patellar dislocation. The main surgical methods were as follows: medial patellofemoral ligament repair or reconstructions, and / or tibial tubercle osteotomy. The patients were excluded in the following situations: osteoarthritis of the patellofemoral joint, traumatic injury of the tibial tubercle, tibial articular fracture, or previous patellar surgery. The control group consisted of 58 patients (60 knees, mean age: 24.3±6.4 years old, female/male: 40/18). They had normal knees or synovial chondroma or a bone or soft-tissue tumor around the knee joint. The patients were excluded in the following situations: ligament injury, tibial articular fracture, or previous surgery. All the people included in this study had CTs and MRIs. Two reviewers reviewed the imaging data including CT and MRI, one experienced sports medicine doctor and one experienced musculoskeletal radiologist. All measurements were made in a blind manner. One month later, 30 samples were randomly selected and two observers repeated the measures to calculated the intra-observer reliability. The imaging data data were analyzed by the software (RadiAnt DICOM Viewer, version 5.5.1).The details were seen in Fig. 1.

### TT-TG measurement

**Fig. 2:**
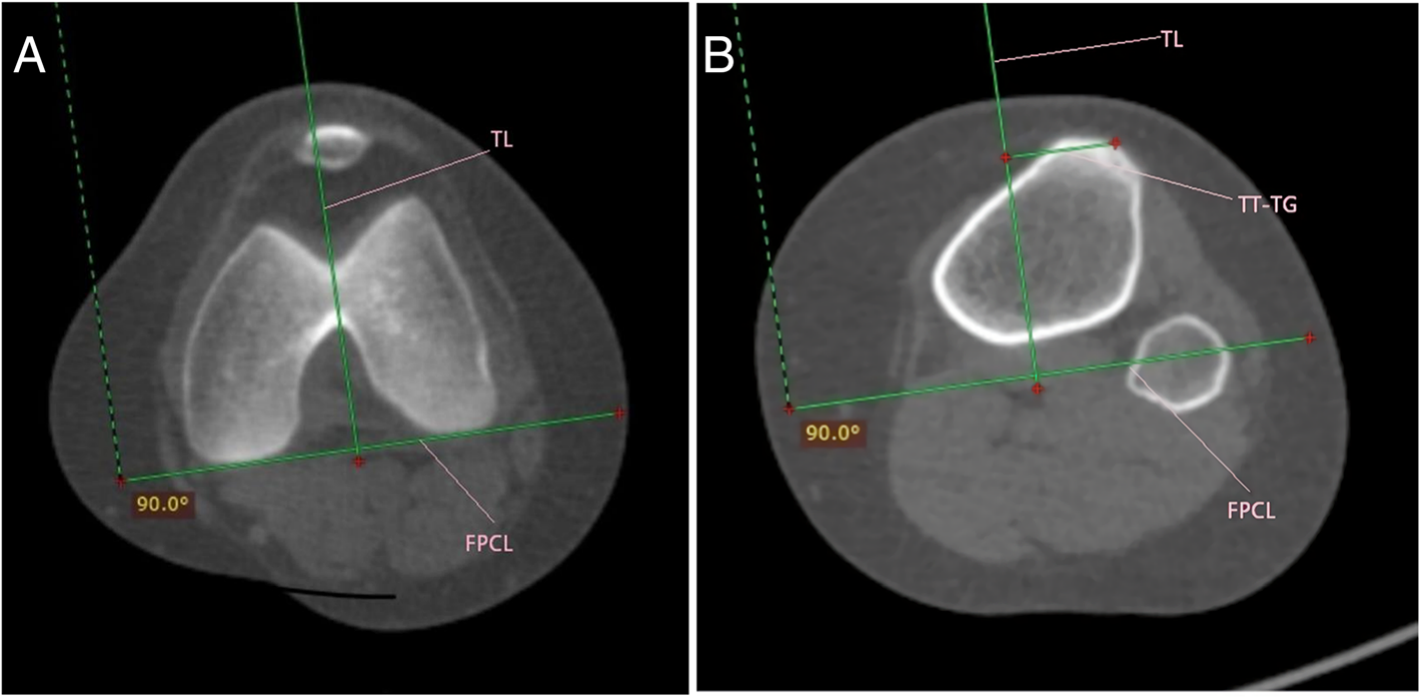
**a**, **b**: Technique for measuring TT-TG distance on Axial computed tomography. FPCL, femoral posterior condylar line; TL, trochlear line; TT-TG, tibial tubercle–trochlear groove distance

**Fig. 3:**
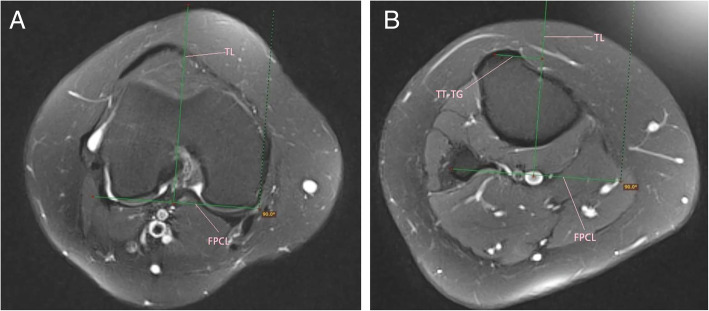
**a**, **b**: Technique for measuring TT-TG distance on Axial magnetic resonance. FPCL, femoral posterior condylar line; TL, trochlear line; TT-TG, tibial tubercle–trochlear groove distance

The method of TT-TG measurement was similar to that of Schoettle et al. [7] Firstly, a tangent line to the posterior femoral condyles called posterior condylar line was drawn. Secondly, a perpendicular line to the posterior condylar line called trochlear line was drawn through the deepest point of the trochlear groove. Thirdly, the trochlear line was transferred to the axial plane with the middle point of the tibial tuberosity. The TT-TG distance was the distance from the middle point of the tibial tuberosity to the trochlear line. Bony landmarks were also used to measure the TT-TG distance on MRI. The details were seen in Figs. 2a, b and 3a, b.

### TT-PCL measurement

**Fig. 4:**
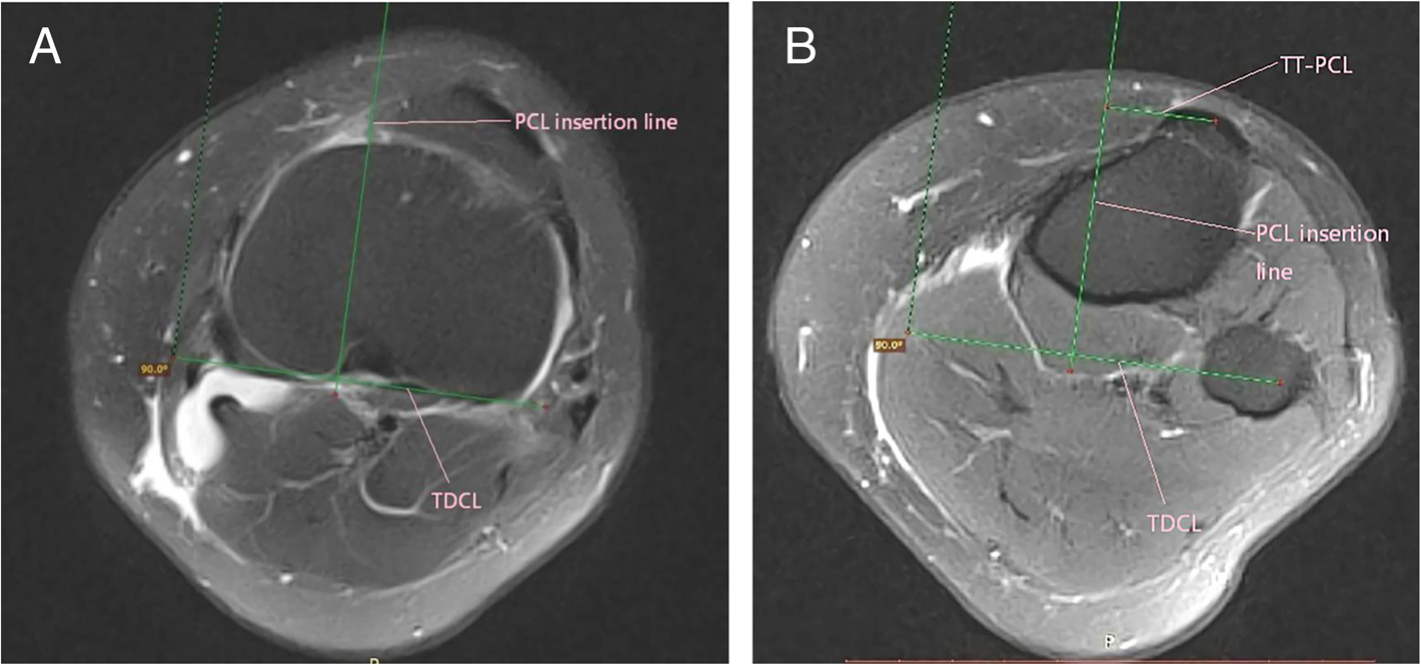
**a**, **b**: Technique for measuring TT-PCL distance on Axial magnetic resonance. TDCL, tibial dorsal condylar line; PCL insertion line, posterior cruciate ligament insertion line; TT-PCL, tibial tubercle–posterior cruciate ligament distance

The method of TT-PCL measurement was similar to that of Anley et al. [12] Firstly, a tangent line to the dorsal tibial condyles on the slice just between the articular cartilage and the proximal head of the fibula called the tibial dorsal condylar line was drawn. Secondly, a perpendicular line to the tibial dorsal condylar line called the tibial plateau line was drawn through the medial border of the PCL. Thirdly, the tibial plateau line was transferred to the axial plane with the midpoint of the inferior patellar tendon insertion at the TT. the TT-PCL distance was the distance from the midpoint of the inferior patellar tendon insertion at the TT to the tibial plateau line. The details were seen in Fig. 4a, b.

### The tibial width

**Fig. 5:**
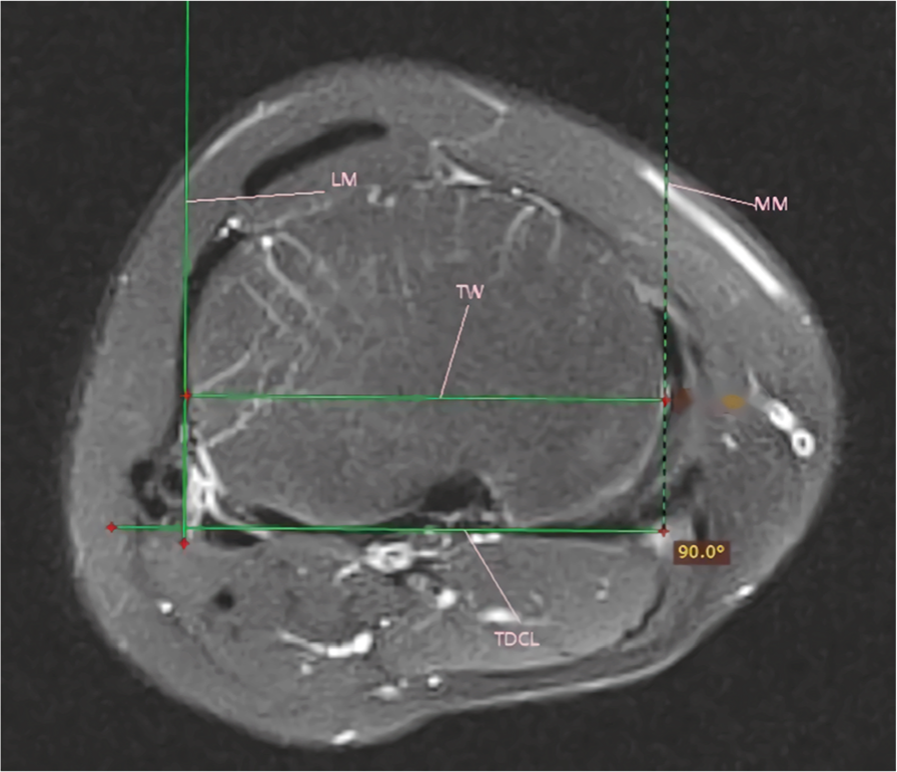
Technique for measuring tibial width on Axial magnetic resonance. MM, the medial margin of the tibial condyle; LM, lateral margin of the tibial condyle;TW, the distance between the medial and lateral margins of the tibial condyle

The method of tibial width measurement was similar to that of Tensho et al. [11] The measurement was performed on the axial plane where the posterior tibial condyles was clearly recognized. Two perpendicular lines to the tibial posterior condylar line were drawn through the medial and lateral margins of the tibial condyle respectively. The tibial width was the distance between two perpendicular lines. The details were seen in Fig. 5.

### CT protocol

The patient’s position was supine with knees straight. A 64–detector row Siemens CT scanner (Siemens Somatom Definition AS+, Germany) was used for examination. The parameters were as follows: matrix (512 × 512), thickness (1-3 mm), scan time (approximately 5 to 10 s), 0-mm skip between slices, FOV (20) and bone kernel.

### MRI protocol

The patient’s position was supine with knees in a coil. A 1.5-T Siemens MRI scanners (Siemens Magntom Avanto, Germany) with axial T2 imaging in all cases was used for examination. The parameters were as follows: fast spin echo, repetition time (TR) (3000 to 5000 milliseconds), echo time (TE) (40 milliseconds), thickness (3 mm), scan time (approximately 3-min), 0.6-mm skip between slices, FOV (16), matrix (352 × 288), number of excitations (NEX) (3), and echo train length (ETL) (8 to 10).

### Statistical analysis

All relevant data were entered in excel and analyzed statistically using IBM SPSS Statics 25 and MedCalc 15.2.2. An unpaired t test was performed to determine significant differences between the different parameters of the case group and the control group. And a paired t test was performed to determine significant differences between TT-TG on CT and TT-TG on MRI. The value of *P*< 0.05 was considered significant. In addition, the Pearson correlation coefficient was used to calculate the correlation between TT-PCL on MRI and the tibial width. The intraclass correlation coefficient (ICC) and Bland-Altman plots was used to evaluate the agreement of multiple measurements by different observers on CT and MRI. An ICC value higher than 0.75 indicates excellent agreement. Receiver operating characteristic (ROC) curves and the area under the ROC curve (AUC) were measured to assess the diagnostic accuracy of different measurements with the cutoffs calculated.

## Results

All patients included in this study had patellar dislocation at least twice, which had a significant negative impact on patients’ life. The mean age of the case group in the study was 19 years (range, 12–38 years). There was a total of 28 male and 75 female patients, and 14 of these patients were with bilateral patellar dislocations. The mean age of the control group was 24 years (range, 13–35 years). There was a total of 18 male and 40 female patients, and two of these patients were with bilateral patellar dislocations.

The mean TT-TG distances of the case group on CT and MRI were 22.1 mm and 19.6 mm, respectively. The mean TT-TG distances of the control group on CT and MRI were 16.8 mm and 14.3 mm, respectively. The intraclass correlation coefficients (ICC) for TT-TG on CT was excellent (0.974; 95% CI, 0.964–0.981; *p*< 0.001). The ICC for TT-TG on MRI was excellent (0.937; 95% CI, 0.900–0.959; *p<* 0.001). The ICC for TT-TG on CT was slightly higher than that on MRI. The mean TT-PCL distance of the case group on MRI was 23.0 mm (range, 14.7–35.1 mm). The mean TT-PCL distance of the control group on MRI was 21.8 mm. The ICC for TT-PCL on MRI (0.712; 95% CI, 0.581–0.798; *p<* 0.001) was worse than that for TT-TG on CT or MRI. The details were seen in Tables 1-2.

**Table 1:** Comparison of control versus case groups. TT–TG: tibial tuberosity–trochlear groove distance. TT–PCL: tibial tuberosity–posterior cruciate ligament distance

parameter	case group				control group				*P* Value
	Mean	SD	Min	Max	Mean	SD	Min	Max	
TT-TG on CT	22.085	3.662	13.35	31.25	16.763	4.223	4.9	30.05	< 0.0001
TT-TG on MRI	19.606	4.426	5.55	33	14.255	4.466	4.95	24.75	< 0.0001
TT-PCL on MRI	23.035	3.846	14.7	35.1	21.843	3.25	14.75	28.2	0.041
Width of tibial plateau	69.134	4.401	61.15	82.55	70.769	5.808	61.35	83.2	0.058

**Table 2:** Results of Intraclass Reliability Calculations. ICC: intraclass correlation coefficient; Interrater Reliability: Reliability between rater A and B for a given imaging modality. Inter-method Reliability: Reliability between CT and MRI for the raters

Reliability	ICC
Interrater	
TT-TG on CT	0.974
TT-TG on MRI	0.937
TT-PCL on MRI	0.712
Inter-method	
Rater A	0.566
Rater B	0.566

**Fig. 6:**
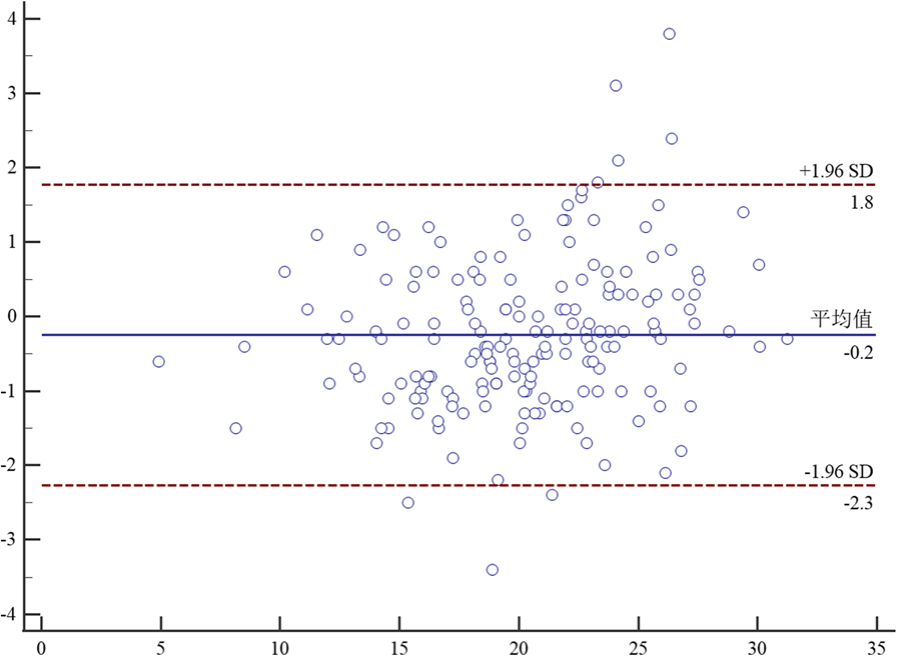
Bland-Altman analysis of interrater agreement between rater A and B for TT-TG on CT

**Fig. 7:**
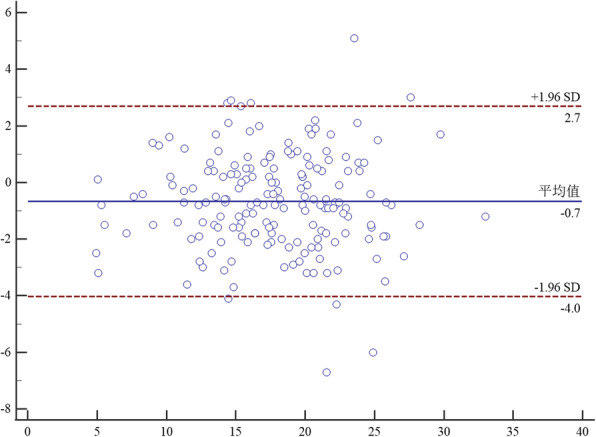
Bland-Altman analysis of interrater agreement between rater A and B for TT-TG on MRI

**Fig. 8:**
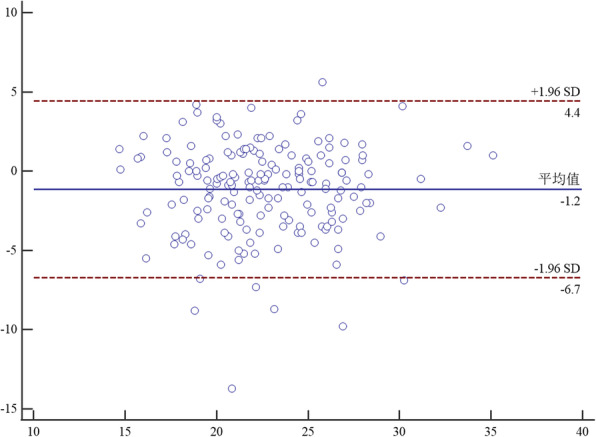
Bland-Altman analysis of interrater agreement between rater A and B for TT-PCL on MRI

Bland-Altman analysis between two raters proved that the mean difference of TT-TG on CT between rater A and B was − 0.2 mm (95% CI, − 2.3-1.8). The mean difference of TT-TG on MRI between rater A and B was − 0.7 mm (95% CI, − 4-2.7).And the mean difference of TT-PCL on MRI between rater A and B was − 1.2 mm (95% CI, − 6.7-4.4). The details were seen in Figs. 6, 7 and 8.

When evaluating the variability of TT-TG on the two imaging modalities (CT and MRI), the ICC for TT-TG on the two imaging modalities evaluated by rater A was 0.566 (95% CI, 0.271–0.731; *p*< 0.001). The ICC for TT-TG on the two imaging modalities evaluated by rater B was 0.566 (95% CI, 0.342–0.708; *p<* 0.001). When comparing the TT-TG distance on CT with that on MRI, the mean TT-TG distance between the two imaging modalities was 2.5 mm (95% CI, 1.9–3.1; *p*< 0.001). There was a significant difference on the TT-TG distance between the two imaging modalities, which indicated the TT-TG distance could not be exchangeable between the two imaging modalities.

The mean tibial width on MRI in the case group was 69.1 mm. The mean width of tibial plateau on MRI in the control group was 70.8 mm (range, 61.4–83.2). There was no significant difference on the tibial width between the case group and the control group (*p*=0.058). Pearson test proved that TT-PCL had a positive significant correlation with the width of tibial plateau (*R=*0.455, *p*< 0.001), which indicated TT-PCL should be considered as an individual parameter in recurrent patellar dislocations.

**Fig. 9:**
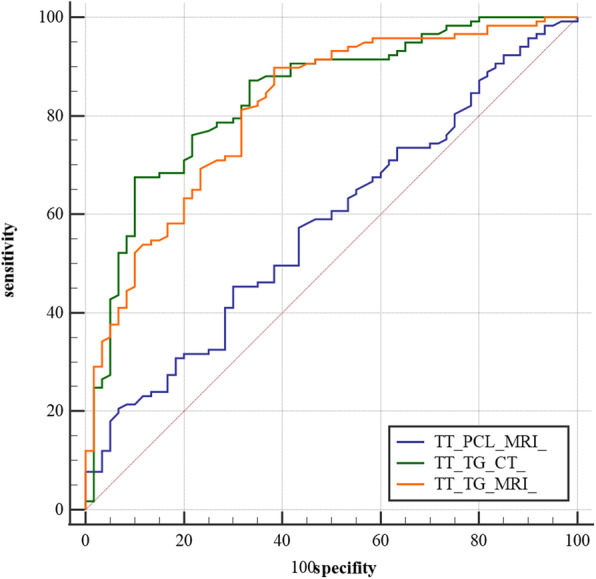
ROC curve showing the AUCs of the TT-TG distance on CT, TT-TG distance on MRI and TT-PCL distance on MRI

For the TT-TG distance on CT, the AUC was 0.838. The sensitivity and specificity were 0.68 and 0.9, respectively. For the TT-TG distance on MRI, the AUC was 0.814. The sensitivity and specificity were 0.9 and 0.62, respectively. For the TT-PCL distance on MRI, the AUC was 0.58. The sensitivity and specificity were 0.45 and 0.7, respectively. The difference of AUC between TT-TG on CT and TT-PCL on MRI was 0.258 (95% CI, 0.175–0.341; *p*< 0.001), which indicated that the diagnostic accuracy of TT-TG on CT was better than that of TT-PCL on MRI. The difference of AUC between TT-TG on MRI and TT-PCL on MRI was 0.234 (95% CI, 0.140–0.328; *p<* 0.001), which indicated that the diagnostic accuracy of TT-TG on MRI was better than that of TT-PCL on MRI. The details of AUC were seen in Table 3 and Fig. 9.

**Table 3:** Diagnostic Performance Parameters of the Measurements

Parameter	AUC	95% CI	*P* Value	Cutoff	Sensitivity	Specificity
TT-TG on CT	0.838	0.775–0.889	< 0.0001	20.6	0.68	0.9
TT-TG on MRI	0.814	0.748–0.868	< 0.0001	14.7	0.9	0.62
TT-PCL on MRI	0.58	0.503–0.653	0.0744	23.3	0.45	0.7

## Discussion

Firstly, a statistically significant difference of the mean TT-TG difference (2.5 mm) and only a fair ICC (0.566) for TT-TG between CT and MRI-proved that TT-TG could not be used interchangeably between the two imaging modalities. Secondly, inter-observer reliability for the TT-PCL measurement (ICC=0.712) was worse than that for the TT-TG on CT (ICC=0.914). The mean TT-TG difference between the case group and the control group on CT was 5.3 mm, which was obviously bigger than the mean TT-PCL difference of 1.2 mm. No doubt that the increase of the distance difference between the case group and the control group was helpful for doctors to distinguish the patients from the normal. Thirdly, Receiver operating characteristic (ROC) curves and the area under the ROC curve (AUC) were measured to assess the diagnostic accuracy of TT-TG and TT-PCL on MRI. The results proved that the diagnostic accuracy of TT-TG on CT (AUC=0.838) were better that of TT-PCL on MRI (AUC=0.58). At last, Pearson test was established to prove that there was a positive correlation between the TT-PCL distance and the width of tibial plateau (*R=*0.455, *p*< 0.001).

A few of studies had reported the ICCs for TT-TG on CT and TT-PCL on MRI. Seitlinger et al. [9] noted that the ICC for TT-PCL was 0.74, which was similar with that obtained in our research (ICC=0.712). Daynes et al. [13] noted the ICC for TT-TG on CT was 0.89, which was similar with that obtained in our research (ICC=0.974). From the points above, we can find that the measurements of the TT-TG distance on CT and the TT-PCL distance on MRI were reliable, and the reliability of the TT-TG distance on CT was better than the TT-PCL distance on MRI.

In terms of the reliability of the two imaging modalities for TT-TG, Camp et al. [14] noted that the ICCs for TT-TG between two imaging modalities were 0.532 for rater A and 0.539 for rater B, respectively. In addition, they found that the TT-TG distance on CT was greater than that on MRI with the mean difference of 2.23 mm. Anley et al. [12] noted that the ICCs for TT-TG between two imaging modalities were 0.54 for rater A and 0.48 for rater B, respectively. In addition, they found the TT-TG distance on CT was greater than that on MRI with the mean difference of 4.16 mm. The results mentioned above were similar to ours. In our research, the ICCs for TT-TG between two imaging modalities were 0.566 for rater A and 0.566 for rater B, respectively. And the TT-TG distance on CT was greater than that on MRI with the mean difference of 2.5 mm (*p*< 0.0001). Considering the low ICC and the significant difference of TT-TG distance between 2 imaging modalities, TT-TG distance between two imaging modalities could not be interchangeable. For the lower values for TT-TG on MRI, it might be caused by increased flexion of the knee with the use of a MRI knee coil [12].

In terms of TT-PCL, Boutris et al. [10] noted that the mean TT-PCL distance of the case group and the control group was 21.1 ± 4.1 and 18.8 ± 4.0 mm, respectively. Daynes et al. [13] noted that the mean TT-PCL of the case group and the control group was 21.62 ± 4.52 and 19.04 ± 4.51 mm, respectively. In terms of TT-TG on CT, Tensho et al. [11] noted that the mean TT-TG distance of the case group and the control group was 19.2 ± 4.0 and 14.3 ± 2.9 mm, respectively. Dejour et al. [5] noted that the mean TT-TG distance of the case group and the control group was 19.8 ± 1.6 and 12.7 ± 3.4 mm,respectively. In our research, the mean TT-TG distance of the case group and the control group was 22.1 ± 3.7 and 16.8 ± 4.2 mm. The mean TT-PCL distance of the case and control group was 23 ± 3.8 and 21.8 ± 3.3 mm. Obviously, the mean TT-TG difference on CT between the case group and the control group was greater than the mean TT-PCL difference, which indicated TT-TG on CT more helpful for doctors to differentiate between patients and the normal.

In addition, Receiver operating characteristic (ROC) curves and the area under the ROC curve (AUC) were established to assess the diagnostic accuracy of TT-TG and TT-PCL. The AUCs of TT-TG on CT and TT-PCL on MRI were 0.838 and 0.58, respectively, which were approximate to those obtained by Tensho et al. (0.84 and 0.66, respectively). It was concluded from the above that the diagnostic accuracy of TT-TG on CT was better than that of TT-PCL on MRI. However, when trochlear dysplasia existed, it was difficult to determine the deepest point of tibial tubercle–trochlear groove. In such a situation, TT-PCL was an ideal choice.

Considering the individual difference, TT-PCL ratio was proposed by Tensho et al. [11] However, few articles proved the correlation between the TT-PCL distance on MRI and the tibial width [15]. In this research, Pearson test was established to confirm the correlation between the TT-PCL distance on MRI and the tibial width. The results indicated that TT-PCL had a positive significant correlation with the width of tibial plateau (*R=*0.455, *p*< 0.001). Considering this factor, TT-TG should be considered as an individual parameter in recurrent patellar dislocations though it is not affected by the flexion of the knees.

The cases included in this study all underwent surgery for patellar dislocation, while the cases in the other studies were described with dislocation more than twice, without illustrating the frequency of dislocation in detail. In clinical setting, the patients choose the surgery as the therapy, always because dislocation so frequently affects the normal life. And TT-TG>20 mm was considered as a standard to make the transferring of tibial tubercle. The patients who underwent surgery for patellar dislocation might be more suitable for the scientific research. This article is limited by the retrospective nature. The position of the knee joint may affect the results of our research.

## Conclusion

A statistically significance and a fair ICC proved that TT-TG could not be used interchangeably between the two imaging modalities. The bigger mean difference between the case group and the control group and better AUC proved that TT-TG on CT might be an indicator more suitable for measuring the lateralization of the tibial tubercle. And TT-PCL should be considered as an individual parameter because of the significant correlation with tibial width.

## Data Availability

The datasets supporting the conclusions of this article are included within the article. Raw data can be requested from the corresponding author.

## Abbreviations

RPD: Recurrent patellar dislocation
CT: Computed tomography
MRI: Magnetic resonance imaging
TT-TG: Tibial tubercle–trochlear groove
TT-PCL: Tibial tubercle–posterior cruciate ligament
ICC: Intraclass correlation coefficient
ROC: Receiver operating characteristic
AUC: Area under the ROC curve
MPFL: Medial patellofemoral ligament
